# Permissive Hypoxemia With Partial Pressure of Oxygen in Arterial Blood (PaO2) at Less Than 50 mmHg: A Case of Successful Ventilator Weaning for COVID-19-Associated Acute Respiratory Failure

**DOI:** 10.7759/cureus.93221

**Published:** 2025-09-25

**Authors:** Hiroshi Adachi, Kwonil Choi, Motohiro Shimizu

**Affiliations:** 1 Intensive Care Department, Ryokusenkai Yonemori Hospital, Kagoshima, JPN

**Keywords:** covid-19 associated acute respiratory failure, individualized management, lung-protective ventilation, permissive hypoxemia, refractory hypoxemia

## Abstract

Permissive hypoxemia is a ventilation strategy that intentionally tolerates mild to moderate hypoxemia to minimize lung injury. Unlike conventional management, which aims for normal oxygen levels, permissive hypoxemia maintains a partial pressure of oxygen in arterial blood (PaO2) of 55-60 mmHg and an arterial oxygen saturation (SaO2) of 88-92%. This approach ensures sufficient oxygen delivery for vital functions while reducing mechanical stress on the lungs from high oxygen concentrations and airway pressures, which can lead to lung injury. This flexible, individualized strategy prioritizes lung protection over achieving absolute oxygenation values.

A woman in her 70s, with COVID-19-associated pneumonia, was admitted with severe acute respiratory failure and required mechanical ventilation. Despite a lung-protective ventilation strategy, her oxygenation remained poor. A permissive hypoxemia strategy was reluctantly initiated, which allowed her PaO2 to drop below 50 mmHg. Under strict monitoring of her symptoms, vital signs, and lactate levels, she was successfully weaned from mechanical ventilation without her respiratory status or other organ systems worsening.

The patient was discharged home walking approximately three weeks after admission, with no other complications. To our knowledge, few reports have described an adult patient who successfully tolerated a PaO₂ below 50 mmHg. This case underscores the importance of a rigorous selection of patients and a comprehensive assessment of their overall condition. It demonstrates that a personalized approach, rather than simply adhering to a high oxygenation target, can lead to successful outcomes in severe respiratory failure, affirming that cautious and well-considered attempts in challenging cases are worthwhile.

## Introduction

Permissive hypoxemia is a ventilatory strategy that intentionally tolerates mild to moderate hypoxemia to minimize lung injury [1-3]. Conventional management aims to maintain partial pressure of oxygen in arterial blood (PaO2) and arterial oxygen saturation (SaO2) within normal ranges. However, the high fraction of inspired oxygen (FiO2) and airway pressures needed to achieve this can lead to oxygen toxicity and ventilator-associated lung injury (VALI).

Permissive hypoxemia aims to maintain a PaO2 of 55-60 mmHg and an SaO2 of 88-92% while ensuring adequate tissue oxygen delivery, as clinically assessed. This ensures adequate oxygen delivery for vital functions while reducing mechanical stress on the lungs, which, in turn, promotes lung repair. As a flexible and individualized approach, it prioritizes lung protection over absolute oxygenation values [1-4]. However, because it is contraindicated in certain pathologies, such as brain injury, careful patient selection and comprehensive systemic management are essential [4].

Patients with COVID-19 can experience a condition known as 'happy hypoxia,' in which they do not feel short of breath or experience difficulty breathing, despite having extremely low blood oxygen saturation (SpO2) [5,6]. Normally, hypoxemia stimulates the brain's respiratory center, causing difficulty breathing, but COVID-19 can disrupt this mechanism. Possible causes include the virus's effect on the nervous system and a decrease in the sensitivity of the respiratory center. As a result, patients may experience a certain level of hypoxia without showing any symptoms, and their condition may worsen without them realizing it [5,6].

We successfully managed a case of acute respiratory failure due to COVID-19 in an adult patient using a permissive hypoxemia strategy, which resulted in a favorable outcome. To our knowledge, few reports have described an adult patient who successfully tolerated a PaO2 of below 50 mmHg.

## Case presentation

**Figure 1:**
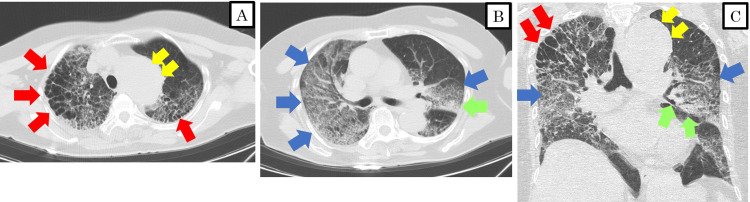
Chest CT images on admission (A, B) Axial view of chest CT imaging; (C) Coronal view of chest CT imaging CT: computed tomography

A woman in her 70s, with a history of hypertension and myocardial infarction, who had been recuperating at home for 10 days after a positive COVID-19 test, developed shortness of breath and was transported to our hospital. She was 158 cm tall and weighed 60.0 kg. Upon arrival, her vitals were Glasgow coma scale (GCS) 15, blood pressure 110/70 mmHg, pulse rate 88/min, and respiratory rate 24/min. Her body temperature was 36.6 ℃, but her SpO2 was critically low at 66%, despite receiving oxygen on a non-rebreather mask at 10 L/min. A chest CT scan revealed severe emphysematous changes, multiple ground-glass opacities, and infiltrative shadows, as well as a distal aortic arch aneurysm (Figure 1). There was no evidence of significant pulmonary consolidation or pulmonary thrombus. Oxygenation did not improve even after the oxygen supply from the reservoir mask was increased to 15 L/min, so she was started on invasive positive pressure ventilation (IPPV) (Table 1). Blood tests showed: WBC 10×10^3^/μl, CRP 11.67 mg/dl, PCT (procalcitonin) 0.12 ng/ml, BNP (brain natriuretic peptide) 63.8 pg/ml, KL-6 (Krebs von den Lungen-6) 1243 U/ml, and SP-D (surfactant protein D) 243 ng/ml (Tables 2, 3).

**Table 1:** Blood gas analysis, mean blood pressure, heart rate, respiratory rate, and ventilator settings on Day 1 pH, potential of hydrogen; PaCO2, partial pressure of arterial carbon dioxide; PaO2, partial pressure of arterial oxygen; HCO3-, bicarbonate; ABE, actual base excess; FiO2, fraction of inspired oxygen; RR, respiratory rate; MAP, mean arterial pressure; HR, heart rate; PRVC, pressure-regulated volume control; Vt, tidal volume; PEEP, positive end-expiratory pressure

	On admission	Post-intubation
Blood gas	Venous	Arterial
pH	7.445	7.2887
PaCO_2_ (mmHg)	38.3	54.7
PaO_2_ (mmHg)	36.8	73.2
HCO_3_^-^ (mmol/L)	26.3	26.1
ABE (mmol/L)	2.2	-1.4
Lac (mmol/L)	1	1.1
FiO_2_ (%)	60-90% (Reservoir mask, 10L/min)	100
RR (/min)	11 (Spontaneous)	20 (Ventilator)
MAP (mmHg)	101	82
HR (/min)	87	74
Ventilator Mode		PRVC
Settings		Vt; 280ml, PEEP; 15 cmH_2_O

**Table 2:** Clinical course of blood test data T-bil, total bilirubin; CK, creatine kinase; LDH, lactate dehydrogenase; Cre, creatinine; CRP, C-reactive protein; WBC, white blood cell; Hb, hemoglobin; Plt, platelet; eGFR, estimated glomerular filtration rate; PCT, procalcitonin

	Day 1	Day 2	Day 3	Day 4	Day 5	Day 6	Day 7	Day 8	Day 9
T-bil (mg/dl)	0.6	0.6	0.6	0.8	0.9	1.3	1.4	1.5	1.0
CK (U/L)	36	101	33	72	63	38	48	63	67
LDH (U/L)	455	483	381	479	629	377	401	325	305
Cre (mg/dl)	0.80	0.76	0.73	0.63	0.56	0.53	0.52	0.63	0.58
CRP (mg/dl)	11.67	12.22	5.46	2.33	1.29	1.85	5.90	4.95	3.44
WBC (×10^3^/μL)	10.0	10.0	15.0	15.1	14.8	14.8	18.7	10.3	16.4
Hb (g/dl)	12.8	13.0	11.9	11.7	12.1	12.7	13.1	12.9	13.0
Plt (×10^3^/μL)	449	394	420	443	489	490	478	521	553
D-dimer (μg/dl)	2.89	6.44	11.28	10.38	11.33	9.06	10.03	6.04	6.44
eGFR (ml/min/1.73m^2^)	53	57	59	69	79	84	86	69	76
PCT (ng/ml)	0.12	0.10	0.06	0.04	0.04	0.07	0.12	0.11	0.08

**Table 3:** Changes in serum levels of KL-6, SP-A, SP-D, and BNP KL-6, Krebs von den Lungen-6; SP-A, surfactant protein-A; SP-D, surfactant protein-D; BNP, brain natriuretic peptide

	Day 1	Day 6	Approx. 3 months later
KL-6 (U/ml)	1243	1306	679
SP-D (ng/ml)	234	302	none
SP-A (ng/ml)	none	63.1	none
BNP (pg/ml)	63.8	69.1	none

After admission to the ICU, she was managed with a lung-protective ventilation strategy. Ventilation was initiated in pressure support ventilation (PSV) mode with a positive end-expiratory pressure (PEEP) of 15 cmH₂O and a pressure support of 10 cmH₂O (Table 4). The resulting tidal volume was 6.87 mL/kg (predicted body weight 52.4 kg). Light sedation was achieved with fentanyl and dexmedetomidine, and her Richmond Agitation-Sedation Scale (RASS) score was maintained between -2 and 0. She also received a three-day course of steroid pulse therapy with methylprednisolone (mPSL), which was then gradually tapered. The patient was started on a continuous heparin infusion in the ICU for anticoagulation to keep her activated partial thromboplastin time (aPTT) between 45 and 75 seconds. The patient did not undergo prone positioning because the chest CT showed no significant pulmonary consolidation. However, her hemodynamics remained stable, and rehabilitation, including sitting upright, was actively performed.

**Table 4:** Daily measurements of body weight, 24-hour urine volume, arterial blood gas analysis, lactate levels, vital signs, ventilator settings, and RASS from Day 2 through Day 8 The patient was admitted to the ICU in the early morning on Day 2 and was managed on a ventilator in pressure support mode. The table summarizes key clinical data, including body weight, 24-hour urine volume, arterial blood gas analysis, lactate levels, vital signs, ventilator settings, and RASS. The data was collected at 6 AM each day from Day 2 to Day 8. Additional data points were also recorded post-extubation on Day 7 and before the patient's discharge from the ICU on Day 8. BW, body weight; pH, potential of hydrogen; PaCO2, partial pressure of arterial carbon dioxide; PaO2, partial pressure of arterial oxygen; HCO3-, bicarbonate; ABE, actual base excess; Lac, lactate; FiO2, fraction of inspired oxygen; PS, pressure support; PEEP, positive end-expiratory pressure; RR, respiratory rate; MAP, mean arterial pressure; HR, heart rate; RASS, Richmond Agitation-Sedation Scale; N/A, not applicable

	Day 2	Day 3	Day 4	Day 5	Day 6	Day 7		Day 8	
	6AM	6AM	6AM	6AM	6AM	6AM	Post-extubation	6AM	Pre-ICU discharge
BW (kg)	59.4	60.3	60.2	61.6	60	58.9	N/A	57.9	N/A
24-hour urine volume (ml)	360	954	1360	1740	2840	2930	N/A	1730	N/A
pH	7.409	7.523	7.538	7.53	7.559	7.564	7.581	7.555	7.548
PaCO_2_ (mmHg)	42.3	33	31.9	31.5	34.3	35.9	33.9	33.4	32.8
PaO_2_ (mmHg)	76.2	52.9	63.9	54.9	49.9	45.3	42.8	46.5	54.1
HCO_3_^-^ (mmol/L)	26.8	27.7	27.1	26.3	30.6	32.5	31.8	29.6	28.5
ABE (mmol/L)	1.8	5.2	4.7	4	8.2	9.8	9.6	7.2	6.2
Lac (mmol/L)	1.2	1.5	1.8	1.9	1.3	1.4	1.4	1.4	1.9
FiO_2_ (%)	70	50	50	50	50	50	35.9	40.7	35.9
PEEP (cmH_2_O)	15	10	10	10	8	7	N/A	N/A	N/A
PS (cmH2O)	10	10	5	5	5	5	N/A	N/A	N/A
RR (/min)	18	18	21	26	18	19	21	22	19
MAP (mmHg)	77	77	87	72	81	68	74	76	64
HR (/min)	67	62	62	68	56	66	80	77	88
RASS	-1	-1	0	0	0	0	0	0	0

Despite mechanical ventilation, the patient's oxygenation remained poor. Given concerns about the potential negative long-term effects of prolonged ventilation, we implemented a strategy of permissive hypoxemia. Her subjective symptoms were monitored closely. Arterial blood gas analysis and lactate levels were measured every six hours at least, while daily morning blood tests evaluated liver, kidney, coagulation function, and creatine kinase (CK) as a screening marker for both cardiac and mesenteric ischemia. During this management, the patient remained asymptomatic with regard to chest and abdominal symptoms. The serial ECG monitor showed no ischemic changes. No significant abnormalities were observed in her vital signs, lactate levels, arterial blood gas analysis, or other laboratory values (Table 4). CK levels consistently remained within the normal range, and based on the absence of both clinical symptoms and ECG abnormalities, we determined that further cardiac-specific enzyme testing with troponin or creatine kinase-MB (CK-MB) was not warranted. Even with her PaO₂ dropping below 50 mmHg on Day 6, no worsening of subjective symptoms, vital signs, lactate levels, or other parameters was observed. This challenging strategy of allowing her PaO₂ to fall below 50 mmHg ultimately led to a successful outcome. The patient was successfully extubated on Day 7 after passing a spontaneous awakening trial (SAT) and a spontaneous breathing trial (SBT). After extubation, she had no dyspnea, and no obvious abnormalities were found in vital signs, lactate levels, arterial blood gas analysis, or laboratory values. On Day 8, she was transferred out of the ICU to continue rehabilitation. She did not experience any other organ dysfunction during her hospital stay and was discharged home on Day 17.

Approximately four months later, she underwent endovascular repair for a distal thoracic aortic aneurysm with no residual respiratory functional abnormalities.

## Discussion

This case presents an instructive example of a patient with refractory hypoxemia due to COVID-19 who was successfully weaned from mechanical ventilation using a challenging permissive hypoxemia strategy, tolerating a PaO₂ of <50 mmHg. This approach highlights the importance of a comprehensive, individualized assessment and a flexible, lung-protective treatment plan over adhering to a uniform oxygenation target.

In mechanical ventilation, the aggressive pursuit of normal oxygenation levels carries the risk of adverse effects such as oxygen toxicity and VALI [1-3]. In cases of refractory hypoxemia, high inspired oxygen concentrations (FiO2) and high PEEP can exacerbate lung damage [1-4]. To mitigate these risks, we adopted a strategy of permissive hypoxemia, which involves tolerating a lower-than-normal PaO2 to minimize VALI. In this case, given the patient's clinical stability and the absence of tissue hypoxia, we tolerated hypoxemia even when the nadir PaO2 dropped to 42.8 mmHg. This approach prioritizes minimizing lung injury over absolute oxygenation values and helps prevent complications such as frailty and secondary infections in elderly patients, ultimately improving patient outcomes and contributing to better preservation of the patient's activities of daily living (ADL).

The pathology of COVID-19-associated acute respiratory failure is distinct from conventional acute respiratory distress syndrome (ARDS), characterized by widespread lung inflammation and damage [5,6]. KL-6 is a glycoprotein made by alveolar type II and bronchial epithelial cells, and it is a biomarker of lung epithelial damage, useful for diagnosing and predicting outcomes in interstitial lung diseases (ILDs), including lung function decline, mortality, and treatment response [7]. SP-D is produced by Clara cells and alveolar epithelial cells and belongs to the C-type lectin superfamily [8]. In healthy lungs, it is found on the surface of alveolar and bronchiolar epithelial cells. When present in the blood or outside the lungs, it serves as a biomarker for various lung diseases such as idiopathic pulmonary fibrosis, ILD, systemic sclerosis, and respiratory infections [9]. In this case, elevated levels of interstitial pneumonia markers, such as KL-6 and SP-D, strongly suggested a coexisting interstitial pneumonia-like condition. Combined with emphysematous changes confirmed by chest CT, these findings suggested that pulmonary fibrosis and decreased lung compliance might progress, making it difficult to improve oxygenation with standard lung-protective ventilation alone. Given this complex pathology, steroid pulse therapy appears to have been effective in suppressing inflammatory changes and slowing disease progression.

A key feature of this case was the patient's apparent state of "happy hypoxia," where they experienced no respiratory distress despite severe hypoxemia. As reported by Akoumianaki et al., this phenomenon can occur in "patients with acute hypoxemic respiratory failure who exhibit normal respiratory muscle function and relatively normal respiratory system mechanics," and is not specific to COVID-19 [10]. This finding has important implications for the management of permissive hypoxemia. Unfortunately, we did not obtain arterial blood gas data prior to intubation. However, upon presentation, the patient was hemodynamically stable and did not complain of dyspnea, and a venous blood gas analysis showed a normal lactate level of 1.0 mmol/L. The patient's muted subjective response to hypoxia, which differed from that of conventional acute respiratory failure, allowed us to safely and successfully implement a strategy of allowing the PaO₂ to fall below 50 mmHg. Even after extubation, the patient remained clinically stable and did not complain of respiratory distress, a testament to the safety and efficacy of this approach in this specific context.

Major randomized controlled trials like the LOCO₂ (Liberal Oxygenation versus Conservative Oxygenation in Acute Respiratory Distress Syndrome) and HOT-ICU (Handling Oxygenation Targets in the ICU) studies have established oxygenation targets for ARDS patients [11,12]. These trials have raised safety concerns regarding lower targets such as those below 90% oxygen saturation. However, this case is unique. Despite severe hypoxemia with a PaO₂ of less than 50 mmHg, the patient maintained normal lactate levels and showed no adverse effects. This highlights that a rigid adherence to universal targets is less important than a comprehensive assessment of the patient's tissue perfusion. The successful outcome in this case suggests that a personalized approach to permissive hypoxemia is crucial and that clinical judgment beyond strict guidelines can lead to positive results.

Strict patient monitoring was essential to safely and effectively implement this strategy. Rather than relying solely on SpO₂ and PaO₂ as indicators of oxygenation, we comprehensively assessed whether the oxygen demand of systemic tissues was being met, which allowed us to manage the patient with a PaO₂ of less than 50 mmHg. Specifically, appropriate sedation and analgesia with fentanyl and dexmedetomidine allowed us to subjectively confirm that the patient had no chest or abdominal symptoms, including dyspnea. In addition to continuous ECG and vital signs monitoring using a vital signs monitor and an invasive arterial pressure monitor, lactate levels and arterial blood gas analysis, which are important indicators of oxygen supply-demand balance, were frequently measured, and blood tests for multiple organ function were also performed daily, as shown in Tables 2 and 4. We monitored creatine kinase (CK) levels not only as an indicator of cardiac function but also as a marker for potential intestinal ischemia. Furthermore, liver function, renal function, and coagulation function were also assessed to confirm the absence of organ damage. We also monitored urine output to ensure adequate renal perfusion. Through such close and comprehensive monitoring, we were able to manage the patient without any serious complications.

Despite the patient's pulmonary function not having fully recovered, a comprehensive assessment of their overall condition supported the decision for early extubation. This bold decision ultimately reduced the risks of disuse syndrome and secondary infection associated with prolonged bed rest, facilitating the patient's early transition to rehabilitation and prompt discharge home.

In conclusion, this case highlights the effectiveness of a flexible, lung-protective strategy for COVID-19-associated acute respiratory failure. By comprehensively assessing the patient's individual pathology and overall condition instead of adhering to a uniform oxygenation target, this challenging approach may be a valid option, potentially leading to better outcomes than those achieved with conventional treatments. However, this report has several important limitations. As a single-case study, the external validity of our findings is limited, and the strategy's safety and efficacy are yet to be established across a broader patient population. We also acknowledge the potential for unmeasured confounders, such as the natural course of the disease or the effects of concurrent treatments like steroids, to have influenced the outcome. Furthermore, while we primarily relied on invasive blood gas analysis, we recognize the potential for measurement errors in non-invasive oximetry. The successful implementation of this approach required strict, comprehensive patient monitoring, and it may not be suitable for all patients. We believe this strategy should be considered a valid but challenging option only for carefully selected patients under close supervision. Furthermore, a flexible approach that allows for a change in strategy based on the patient's condition was essential. Further case studies and validation are needed to establish its safety and efficacy more broadly.

## Conclusions

This case demonstrates the safe and successful application of a highly challenging, permissive hypoxemia strategy, tolerating a PaO₂ of <50 mmHg, in a patient with COVID-19-associated acute respiratory failure. This approach led to successful weaning from mechanical ventilation and highlights the importance of a comprehensive, individualized assessment of a patient's overall condition, rather than strict adherence to a uniform oxygenation target.

We intentionally tolerated hypoxic conditions to minimize the risks of VALI and oxygen toxicity due to aggressive ventilator settings and to prevent complications such as frailty due to long-term mechanical ventilation. This bold strategy was made possible by strict systemic monitoring, including the patient's symptoms, vital signs, and lactate levels, which confirmed adequate tissue oxygenation despite low PaO₂. Furthermore, a flexible decision for early extubation was made despite insufficient oxygenation, which ultimately prevented complications associated with prolonged bed rest and led to a favorable outcome. In conclusion, this case suggests that for refractory acute respiratory failure, a patient-specific, lung-protective strategy, rather than a one-size-fits-all approach, may lead to better outcomes.
